# Effect of *Buah Merah* (*Pandanus conoideus* Lamk.) Extract Supplementation on the Density and Apoptosis of Photoreceptor and Retinal Ganglion Cells in a Diabetic Rat Model

**DOI:** 10.3390/life15111754

**Published:** 2025-11-14

**Authors:** Andi Muhammad Ichsan, Susan Waterina Salle, Itzar Chaidir Islam, Subehan Lallo, Andi Alfian Zainuddin, Budu Mannyu, Habibah Setyawati Muhiddin

**Affiliations:** 1 Department of Ophthalmology, Faculty of Medicine, Hasanuddin University, Makassar 90245, Indonesiaitzarislam@unhas.ac.id (I.C.I.);; 2 Research Unit of Hasanuddin University Hospital, Makassar 90245, Indonesia; 3 Department of Pharmaceutical Science, Faculty of Pharmacy, Hasanuddin University, Makassar 90245, Indonesia; 4 Department of Public Health, Faculty of Medicine, Hasanuddin University, Makassar 90245, Indonesia

**Keywords:** diabetic retinopathy, *buah merah* extract, retinal cell density, retinal cell apoptosis

## Abstract

Diabetic retinopathy (DR) is a microvascular disorder of the retina due to diabetes mellitus (DM). Natural products are widely used to prevent and treat various diseases caused by DM. This study aims to evaluate the effect of *buah merah* (*Pandanus conoideus* Lamk.) extract on retinal density and apoptosis in a diabetic rat model. A total of 30 male rats (*Rattus norvegicus*) weighing 120–150 g were induced with diabetes using alloxan and divided into five groups: group 1 (normal control), 2 (diabetic control), 3 (diabetes + 1 mL *buah merah* extract), 4 (1.5 mL), and 5 (2 mL). *Buah merah* extract, equivalent to 12 mg total carotenoids, 10 mg total tocopherols, 1.348 mg alpha-tocopherol, and 3.4 mg beta-carotene, was administered for 14 days. Retina was examined using hematoxylin and eosin (HE) staining for photoreceptor and retinal ganglion cell density, and immunohistochemistry (IHC) of Caspase-3 for apoptosis. The results showed that group 3 had photoreceptor and retinal ganglion cell densities close to normal, with photoreceptor density values of 722.52 ± 147.56 and ganglion 18.73 ± 5.61. The post hoc test confirmed a significant protective effect of *buah merah* extract in group 3 (*p*-value 0.014). However, *buah merah* extract was found to maintain photoreceptor and retinal ganglion cell density, but no significant inhibitory effect was observed on photoreceptor or retinal ganglion cell apoptosis. Further studies are needed to better understand the mechanism and potential therapeutic effect of *buah merah* extract.

## 1. Introduction

Diabetes mellitus is a rapidly increasing global health burden, and diabetic retinopathy stands out as one of its most serious microvascular complications. It is estimated that around 22% of people with diabetes worldwide develop DR, while approximately 6% suffer from vision-threatening diabetic retinopathy (VTDR) [1]. In 2020, this accounted for about 103 million adults affected by DR, with projections indicating that the number will rise to over 160 million by 2045, in line with the global diabetes epidemic [2,3].

Regional prevalence further demonstrates the magnitude of this problem. In the United States, DR is estimated to affect 26.4% of diabetic patients, while in India, prevalence studies report 16.9% in patients under 50 years, with similar rates in older age groups ranging from 18% to 19%. Such findings confirm that DR is not only widespread but also consistently present across various populations and age brackets, highlighting its role as a major cause of preventable blindness worldwide (Feenstra, Yego & Mohr, 2013; Baptista, Paniagua & Tavares, 2010) [4,5,6].

These prevalence data underscore the pervasive impact of DR and the urgent need for preventive and therapeutic strategies. Conventional management focuses on systemic control of diabetes, but adjunctive approaches such as antioxidants—including tocotrienol-rich vitamin E (Tocovid)—have shown potential in reducing disease progression and protecting retinal health [7]. This combination of systemic management and emerging therapies offers hope in addressing the global burden of DR [3,8]. The recent literature also emphasizes the neuroprotective and antioxidant pathways involved in diabetic retinopathy progression, further underscoring the relevance of antioxidant-based interventions such as Pandanus conoideus extract [9,10,11].

Diabetic retinopathy is a microvascular disorder of the retina resulting from the long-term effects of diabetes mellitus [12]. This disorder is classically described by progressive changes in the microvasculature leading to retinal ischemia, neovascularization, changes in retinal permeability, and macular edema. According to recent studies, retinal neurodegeneration is a crucial trait associated with disease progression, and early retinal nerve injury precedes microangiopathy. Meanwhile, oxidative stress is a causative factor in increased capillary permeability, damage to the blood–retinal barrier (BRB), retinal capillary cell apoptosis, microvascular abnormalities, and neovascularization [9].

DR is generally managed through strict glycemic control and regular ophthalmologic monitoring. A more severe DR leads to a tighter control schedule. In patients with severe non-proliferative diabetic retinopathy (NPDR), examinations can be performed every 2–4 months. The first-line treatment for patients with PDR is anti-vascular endothelial growth factor (anti-VEGF). However, this treatment is only effective in treating cases of diabetic macular edema (DME). Current DR treatment is still focused on treating advanced DR, often after permanent damage has occurred, necessitating preventive care or early pathology management. According to the results of a previous study, dietary supplements containing antioxidants are associated with inhibiting retinal metabolic abnormalities, reducing apoptosis, and promoting pericyte recovery [13].

Several vitamin supplements have been widely reported to be beneficial in DR. This includes vitamin E (alpha-tocopherol), an essential micronutrient and fat-soluble antioxidant with a proposed role in protecting tissues from uncontrolled lipid peroxidation [14]. The vitamin also contains essential protein functions and gene-modulating effects. In a meta-analysis, alpha-tocopherol supplementation was found to reduce total cholesterol (TC) levels and increase high-density lipoprotein cholesterol (HDL-C) when administered for ≥12 weeks [15].

*Buah merah* (*Pandanus conoideus* Lamk.) is a natural plant native to Indonesia, offering numerous benefits. This fruit is known to have a high content of monounsaturated fatty acids (oleic acid) and various biochemical compounds, such as carotene, cryptoxanthin, tocopherol, phenolic compounds, and flavonoids, with potential applications in functional foods and medicine. The chemical composition of *buah merah* varies, including 3.12–6.48% average protein value, 11.21–30.72% fat, 43.86–79.66% carbohydrates, 3.78–21.88 mg/100 g vitamin C, 0.97–3.14 mg/100 g vitamin B1, 0.53–1.11% calcium (Ca), 8.32–123.03% iron (Fe), 0.01–0.33% phosphorus (*p*), 333–3309 ppm total carotenoids, and 964–11,918 ppm tocopherols [16]. *Buah merah’s* oil is traditionally used by indigenous people, based on experiential knowledge, as a natural medicine for various diseases, including cancer, rheumatoid arthritis, stroke, and HIV/AIDS [17].

Red fruit oil has also been investigated for its potential benefits beyond glycemic control. Experimental studies suggest that its bioactive compounds, particularly carotenoids and tocopherols, may support renal protection in conditions associated with diabetes. In nephropathy models, red fruit oil demonstrated antihyperglycemic properties that correlated with improvements in kidney function, indicating its possible therapeutic role in diabetic nephropathy and related metabolic complications, also including its anti-inflammatory and hepatoprotective effects, to highlight its broad therapeutic potential and rationale for use in ocular disease [18]. Despite these limitations, the interest in red fruit oil as a complementary intervention persists, with future directions likely focusing on refining dosage, formulation, and understanding its mechanisms of action in both metabolic and oxidative stress-related disorders [19].

Therefore, this study aims to evaluate the effect of *buah merah* extract (*Pandanus conoideus Lamk.*) on retinal density and apoptosis in a diabetic rat model. The results are expected to serve as the basis for tracing the effect of administering *buah merah* extract on the protection of retinal cells in cases of DR.

## 2. Materials and Methods

### 2.1. Study Design and Experimental Setting

An experimental post-test-only control group design was adopted. The study was conducted at the Animal Laboratory and the Anatomical Pathology Laboratory, Faculty of Medicine, Hasanuddin University. The procedures followed the Institutional Animal Care and Use Committee (IACUC) guidelines [20], and all animal handling complied with ethical approval from the Hasanuddin University Ethics Committee (Approval No. 92/UN.4.6.4.5.31/PP36/2024 on 20 February 2024).

### 2.2. Experimental Animals (Pre-Treatment Phase)

Thirty male Wistar rats (*Rattus norvegicus*) weighing 120–150 g, without any genetic modification, were included. Animal allocation into groups was performed using a simple randomization method. The investigators conducting histological and immunohistochemical analyses were blinded to the treatment groups to minimize observer bias. Animals were acclimatized for one week under standard housing conditions, provided with regular chow and water ad libitum. Any animal that died during adaptation was excluded from the study. After adaptation, rats were randomly divided into five groups (n = 6 per group): group 1—normal control; group 2—diabetic control; group 3—diabetic + 1 mL *buah merah* extract; group 4—diabetic + 1.5 mL *buah merah* extract; and group 5—diabetic + 2 mL *buah merah* extract. Each 1 mL of *buah merah* extract was equivalent to 12 mg total carotenoids, 10 mg total tocopherols, 1.348 mg α-tocopherol, and 3.4 mg β-carotene.

### 2.3. Induction of Diabetes (Treatment Initiation Phase)

Hyperglycemia was induced using Alloxan (Sigma-Aldrich, St. Louis, MO, USA, Cat. No. A7413, CAS No. 2244–11-3) at a dose of 120 mg/kg body weight administered intraperitoneally. Blood glucose levels were monitored using an Autocheck^®^ multi-monitoring system via tail-vein sampling at three time points: before Alloxan induction (baseline), three days after injection (checkpoint), and one day before termination (post-treatment). Blood glucose levels were categorized as follows: normal (75–150 mg/dL), mild diabetes (150–200 mg/dL), and severe diabetes (200–400 mg/dL). The random blood glucose threshold used to confirm diabetes was ≥200 mg/dL, in accordance with the standard criteria for rodent diabetic models [21].

### 2.4. Preparation of Pandanus Conoideus (Buah Merah) Extract

The extract was prepared and standardized at the Phytopharmaceutical Laboratory, Hasanuddin University. Fully ripe fruits were washed, cut, crushed, and underwent hot extraction. The pulp was dried to retain 20–25% of its weight and macerated using 96% ethanol for 24 h with intermittent stirring every 8 h. Filtrates were collected, concentrated under reduced pressure at 45–50 °C using a rotary evaporator, and evaporated on a water bath to remove residual solvent. Approximately 150 mL of thick ethanolic extract was obtained from 250 g of dried fruit pulp and stored in airtight, light-protected containers. The chemical composition of the extract was verified using spectrophotometric analysis to quantify carotenoid and tocopherol content, following a validated protocol for ethanolic plant extracts. Each 1 mL of extract contained 12,000 ppm (12 mg) total carotenoids, 10,000 ppm (10 mg) total tocopherols, 3581 ppm (3.4 mg) β-carotene, 1460 ppm (1.5 mg) β-cryptoxanthin, 1368 ppm (1.36 mg) α-tocopherol, 74.6% oleic acid, 8% linoleic acid, and 2.1% decanoate.

### 2.5. Administration of Buah Merah Extract (During-Treatment Phase)

The extract was administered orally via gavage once daily for 14 consecutive days at doses of 1 mL, 1.5 mL, and 2 mL, equivalent to approximately 166–200 mg/kg BW, 250–300 mg/kg BW, and 333–400 mg/kg BW, respectively. The doses were selected based on previous preclinical studies demonstrating antioxidant efficacy and safety of *buah merah* extract within a comparable range, while also enabling evaluation of dose–response characteristics [22]. Treatment began immediately after confirming post-Alloxan hyperglycemia to ensure a consistent baseline diabetic status before intervention. One animal in group 5 died during treatment due to non-treatment-related causes.

### 2.6. Sample Termination and Tissue Collection (Post-Treatment Phase)

After 14 days of extract administration, all rats were terminated under ether anesthesia. The eyes were enucleated by transecting the optic nerve and fixed in 10% neutral-buffered formalin for histopathological evaluation. Body weight and blood glucose were measured at pre-Alloxan, post-Alloxan, and pre-termination timepoints.

### 2.7. Histopathological and Immunohistochemical Evaluation

Retinal sections were stained with hematoxylin–eosin (HE) to assess the density of photoreceptor and retinal ganglion cells at 400× magnification. Counting of cell nuclei in three randomly selected, non-overlapping fields of view within the central retina to obtain a representative average density and minimize selection bias. Apoptosis was evaluated by immunohistochemical staining using monoclonal anti–Caspase-3 antibodies (Sigma-Aldrich, Cat. No. C9598, St. Louis, MO, USA) and Invitrogen antibody (Thermo Fisher Scientific, Waltham, MA, USA).. For photoreceptor cells, apoptosis was assessed semi-quantitatively using a modified Immunoreactivity Scoring System (IRS): (1) negative <10%, (2) Low 10–20%, and (3) high >20%. For retinal ganglion cells, apoptosis was assessed quantitatively as the mean ± SD of Caspase-3-positive cells [22,23].

### 2.8. Statistical Analysis

Data normality was tested with the Shapiro–Wilk test. Normally distributed data were analyzed using One-Way ANOVA followed by LSD post hoc tests, while non-parametric data were analyzed using Kruskal–Wallis and Mann–Whitney tests. All analyses were conducted using SPSS^®^ version 24.0, with statistical significance set at *p* < 0.05. Results were presented as tables, graphs, and descriptive summaries.

## 3. Results

An experimental study with a post-test only control group method was conducted to determine the effect of *buah merah* (*Pandanus conoideus* Lamk.) extract on the retinal layer of diabetic rat models.

### 3.1. Blood Sugar Profiles in Experimental Animals

Blood sugar levels were measured at three time points (pre-alloxan, post-alloxan, and pre-termination). The normal range of blood sugar in normal rats was 75–150 mg/dL. In group 1, the average random blood glucose in the study was in the normal range (<150 mg/dL) with a *p*-value of 0.65, which showed no significant difference. Meanwhile, for diabetes-induced rats, all four groups had levels >150 mg/dL after alloxan injection. There was a significant difference in random blood glucose levels between diabetes groups 2, 3, 4, and 5, with a *p*-value of <0.05 (Table 1).

### 3.2. Photoreceptor Cell Density and Retinal Ganglion Cell Density in Experimental Animals

**Figure 1:**
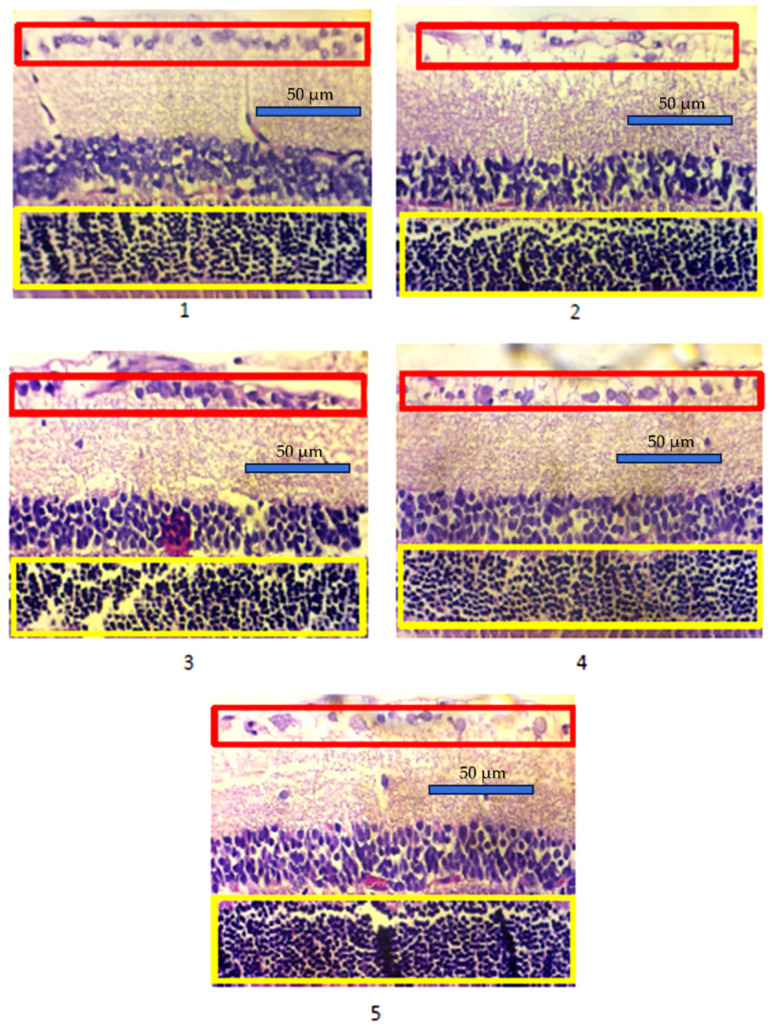
Representative results of retinal photoreceptor and ganglion layers density with HE staining. Groups 1 (normal control), 2 (diabetic control), 3 (diabetic + 1 ml *buah merah* extract), 4 (diabetic + 1.5 ml *buah merah* extract), and 5 (diabetic + 2 ml *buah merah* extract) are shown; the yellow square is the density of photoreceptor cells, and the red square is the density of retinal ganglion cells of the experimental animals.

The density assessment of photoreceptor cells in the retina was carried out by counting the number of photoreceptor nuclei in the outer nuclear layer. Similarly, the density of ganglion cells was assessed by counting the number of ganglion nuclei averaged over 3 fields of view (approximately 0.045 mm^2^ per field of view). Figure 1 shows a comparison of the average photoreceptor and retinal ganglion cell densities of each group. The average density of photoreceptor cells and ganglion cells in the experimental animals is shown in Table 2. The highest and lowest photoreceptor cell densities were found in group 1 (787.97 ± 18.78) and 2 (572.93 ± 45.40), respectively. In the treatment groups of *buah merah* extract, the photoreceptor cell density value in group 3 was close to group 1 (722.52 ± 147.56), while groups 4 and 5 had similar photoreceptor cell density values (605.03 ± 77.46 and 604.28 ± 75.23). One-Way Analysis of Variance (ANOVA) obtained a significant difference between group 4, with a *p*-value of 0.001. The average density of ganglion cells was highest in group 1 (22.77 ± 2.63) and lowest in group 2 (13.20 ± 2.31). The average density of ganglion cells in group 3 was close to the value in groups 1 and 2 (18.73 ± 5.61). Similarly, the average density of ganglion cells in groups 4 and 5 was close to group 2 (14.86 ± 3.28 and 13.84 ± 3.13). ANOVA test showed a *p*-value of 0.001, suggesting a significant difference in retinal ganglion cell density between groups.

**Figure 2:**
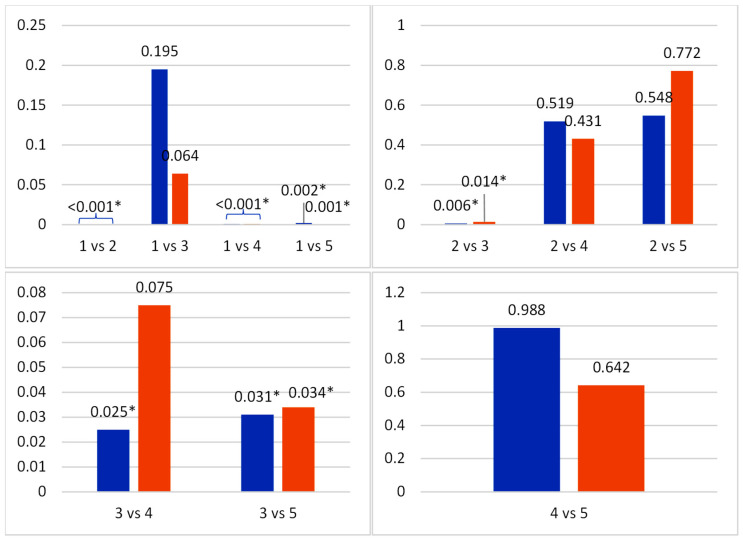
Multivariate analysis of photoreceptor (blue bar) and retinal ganglion cell densities (red bar) (groups = 1: normal control; 2: diabetic control; 3: diabetic + 1 mL extract; 4: diabetic + 1.5 mL extract; 5: diabetic + 2 mL extract), * = sig *p* < 0.05.

Post hoc analysis was carried out after obtaining significant results using a Least Significant Difference (LSD) post hoc test, as shown in Figure 2. Post hoc analysis showed significantly higher Caspase-3 expression in the diabetic control compared to the normal group (*p* < 0.001). The 1 mL extract group demonstrated a significant reduction versus diabetic control (*p* < 0.05), whereas the 1.5 mL and 2 mL groups showed no significant difference from the diabetic control (*p* > 0.05). No treatment group differed significantly from the normal group.

### 3.3. Apoptosis of Photoreceptor Cells and Ganglion Cells in Experimental Animals

**Figure 3:**
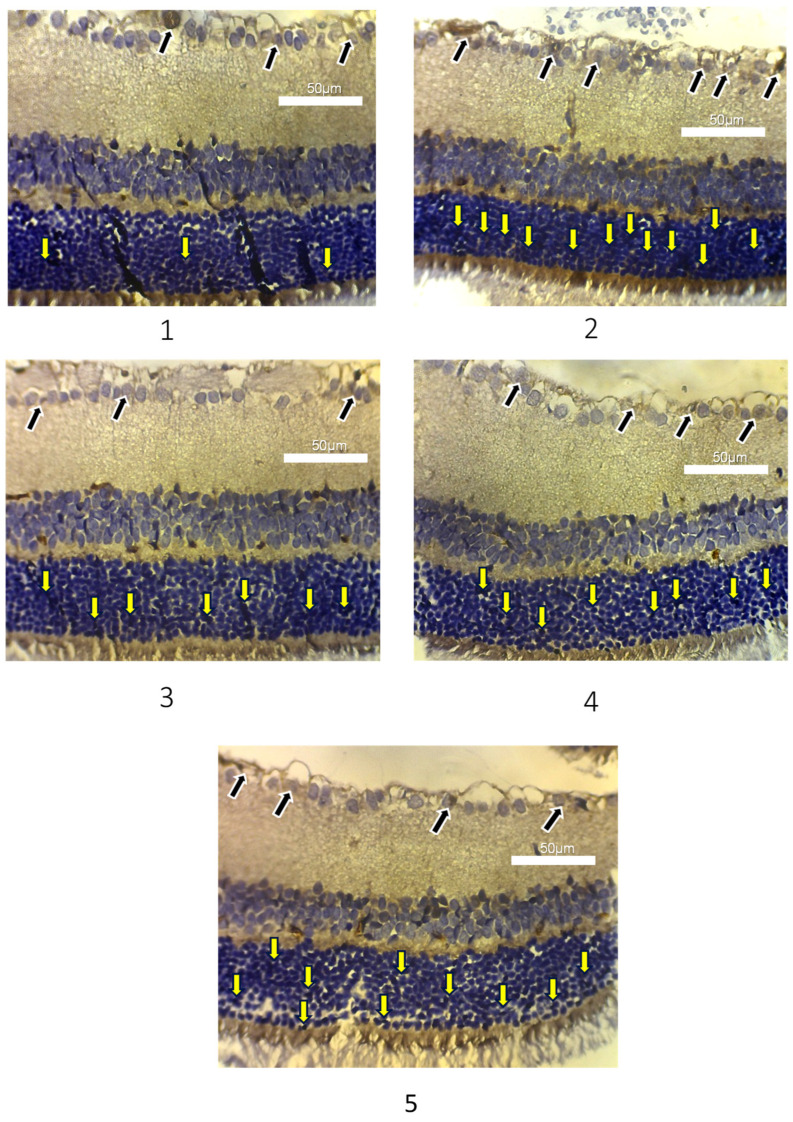
Representative results of IHC caspase-3 expression in the retinal layers. Groups 1 (normal control), 2 (diabetic control), 3 (diabetic + 1 ml *buah merah* extract), 4 (diabetic + 1.5 ml *buah merah* extract), and 5 (diabetic + 2 ml *buah merah* extract) are shown; the yellow arrow shows caspase-3 expression in photoreceptor cells, and the black arrow shows caspase-3 expression in retinal ganglion cells.

Apoptosis assessment of photoreceptor cells and retinal ganglion cells in experimental animals was measured based on the amount of caspase-3 expression as a marker of cell apoptosis (Figure 3). The calculation of caspase-3 expression was assessed semi-quantitatively, after counting the photoreceptor cells stained with brown pigment, and then categorized into three groups: negative, low, and high groups. In Table 3, group 1 showed the majority of photoreceptor Caspase-3 expression in the negative category (five out of six), followed by 1 low category. Group 2 showed the highest Caspase-3 expression in the low category (four out of six), with two high and none negative. In the treatment groups, group 3 showed an even distribution between the negative and low categories, with none high. Group 4 showed four low, two negative, and no high cases, consistent with Table 3. The majority of Caspase-3 expression in group 5 was in the low category (five out of six), with only one negative. The results of the Kruskal–Wallis nonparametric test showed a difference between photoreceptor Caspase-3 expression in each treatment group, with a *p*-value of 0.020.

**Figure 4:**
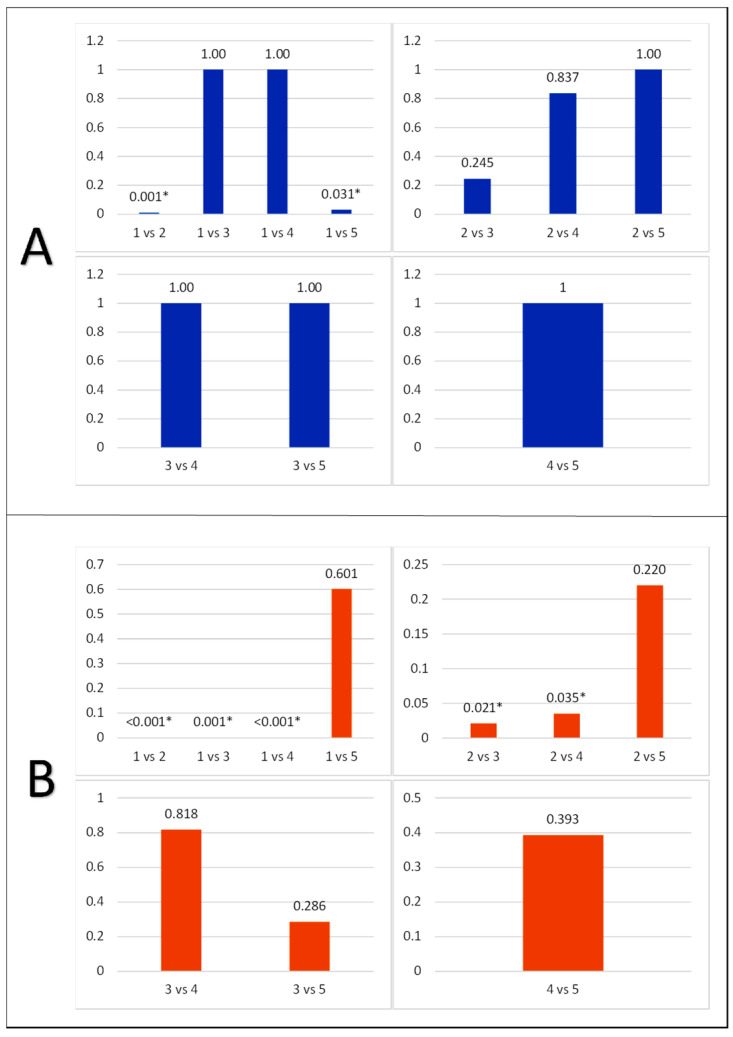
Multivariate analysis of caspase-3 expression on photoreceptor (**A**) and retinal ganglion cells (**B**) (groups = 1: normal control; 2: diabetic control; 3: diabetic + 1 mL extract; 4: diabetic + 1.5 mL extract; 5: diabetic + 2 mL extract). The asterisk (*) in the figure legend, indicating that it represents a statistically significant difference (*p* < 0.05).

In Table 4, group 1 exhibited the lowest average caspase-3 expression in ganglion cells (23.50 ± 3.50), while group 2 showed the highest (46.33 ± 6.05). Caspase-3 expression in ganglion cells in groups 3, 4, and 5 was 37.50 ± 5.01, 38.33 ± 9.67, and 41.60 ± 4.67, respectively. One-Way ANOVA test showed a *p*-value of 0.001, suggesting a significant difference in the average caspase-3 expression in each group. A post hoc test was conducted on the expression of caspase-3 in photoreceptor cells using the Mann–Whitney test (Figure 4). The result showed that the expression of caspase-3 in photoreceptor cells in groups 1 and 2 was significantly different (0.010). In groups 3, 4, and 5, this expression was not significantly different from 1, with the same *p*-value (1.000, 1.000, and 0.601). Groups 3 and 4 did not differ significantly from 2, with *p*-values of 0.245 and 0.837, respectively. However, group 5 showed no significant difference from 2, with a *p*-value of 1.000.

## 4. Discussion

This study aimed to determine the effects of *buah merah* on the density and apoptosis of photoreceptor and retinal ganglion cells in diabetic rat models. The experimental animals were diabetic Wistar rats with a total of 30 samples, with 1 rat dropping out during the treatment duration. Based on cage-side clinical observations and necropsy findings, the death was not attributable to direct toxicity of *buah merah* extract, but rather to aggressive behavior and cannibalism, which are well-documented phenomena in rodent colonies. Although the 14-day observation period may seem limited, this timeframe was intentionally selected to capture the early neurodegenerative and apoptotic phases of diabetic retinopathy rather than its advanced vascular manifestations. Early retinal stress responses can be detected within two weeks post-induction in rodent models, as supported by previous studies [24,25,26].

In our study, the deceased rats exhibited external injuries consistent with cannibalistic attacks by cage-mates, supporting this as the primary cause of mortality. In this study, every 1 mL of *buah merah* extract is equivalent to a total of 12 mg carotenoids, 10 mg total tocopherol, 1.348 mg alpha tocopherol, and 3.4 mg beta-carotene. The mechanism of apoptosis in DR played an important role in understanding the progression of the disease and is also expected to help in promotive, preventive, and curative efforts. Several studies have shown the role of antioxidants in DR, both synthetically obtained antioxidants and antioxidants derived from natural plants [3]. Furthermore, several studies have investigated the effects of natural plants on humans and experimental animals, showing their potential to prevent hyperglycemia and inhibit diabetes-related complications, including nephropathy, neuropathy, and retinopathy. Studies related to the role of natural plants in DR include *Annona muricata*, *Camellia sinensis*, *Curcuma longa*, *Ginkgo biloba*, *Paenioa suffruticosa*, *Pinus pinaster*, *Trigonella foenum-graecum*, *Vaccinium myrtillus*, and *Vitis vinifera* [27].

*Buah merah* from Papua is a known natural plant in Indonesia with many health benefits, such as playing a role in diabetes, hypercholesterolemia, malaria, HIV, cancer, and others. The results of this study showed that the weight of the experimental animals differed significantly in the normal group because there was an increase in body weight. Meanwhile, in the group of diabetic rats with treatment in groups 3, 4, and 5, there was an increase in body weight post-Alloxan induction on the third day, followed by a decrease pre-termination. In the diabetic control group, no significant changes in body weight were found in pre-Alloxan, post-Alloxan, and pre-termination. The hyperglycemic condition in diabetic rats during the 2-week treatment duration has caused weight loss. A study by Febriyanti et al. (2011) reported that weight loss in a group of diabetic rats was caused by a lack of insulin. This insulin decreases the glucose entering the cells, causing the metabolism of protein and fat into energy, thereby decreasing the fat reserves in diabetic rats. In contrast to the result, a previous study reported an increase in body weight of diabetic rats given *buah merah* extract on the fifth day. The nature of alpha tocopherol in *buah merah* acts as an antioxidant that fights free radicals from unsaturated fatty acids, leading to the protection of fat from ROS [28]. The potential of *buah merah* extract in reducing blood sugar levels is related to the function of the active compounds tocopherol, carotene, beta carotene, and ascorbic acid. These compounds inhibit the work of the alpha-glucosidase enzyme, which degrades and converts carbohydrates into glucose. When the function of the enzyme is inhibited, there is a decrease in blood sugar levels, thereby returning to normal [18].

In this study, hyperglycemia was found in the diabetic group without treatment, and also in the *buah merah* extract treatment for 14 days. This was evident in the average random blood glucose level of >200 mg/dL before rats were terminated. Group 2 (diabetic control) had the highest random blood glucose level at pre-termination, with a value in the random blood glucose category of 418.83 ± 144.51. The diabetic group with treatment had a random blood glucose range of 382.17 ± 49.94 mg/dL, 375.83 ± 66.79 mg/dL, and 361.60 ± 62.65 mg/dL before termination. All diabetic groups had random blood glucose levels in the range of 200–400 mg/dL, showing that experimental animals had severe diabetes mellitus. Therefore, the effect of *buah merah* extract dose did not succeed in reducing the hyperglycemia of rats. This result proves that our intention was not to model the full clinical spectrum of diabetic retinopathy (which evolves over weeks to months) but to interrogate early retinal stress and apoptosis following hyperglycemia.

A literature review by Ighodaro et al. (2018) reported the inconsistency factor of alloxan induction material in diabetic rat models. There was a duration of hyperglycemia stability of less than 1 month, and during this period, there was no adequate strength to evaluate the drug [24]. The result was also consistent with the report of Kowluru et al. (2003) that the effects on the retinas of diabetic rat models given a combination of antioxidants ascorbic acid 1 g/kg; trolox 500 mg/kg; dl-alpha tocopherol acetate 250 mg/kg; n-acetyl cysteine, 200 mg/kg; beta carotene 45 mg/kg; and selenium, 0.1 mg/kg did not affect the severity of hyperglycemia, glycated hemoglobin levels, and body weight in the treatment group. The study reported that there was still a possibility of a combination of antioxidants being given to diabetic rats that worked through mechanisms other than those related to correcting oxidative stress. For example, alpha tocopherol can normalize the increase in PKC activity induced by diabetes through its effect on the accumulation of diacylglycerol [25].

Active compounds that play a role in *buah merah* include carotenoids, total tocopherol, beta-carotene, B-cryptoxanthin, alpha tocopherol, oleic acid, linoleic acid, and others. Some of these compounds have been known to be beneficial in DR. Carotenoids are a large group of organic and lipophilic pigments, produced by plants, algae, and some bacteria and fungi, used in the treatment of Age-Related Eye Disease Studies (AREDSs) at a dose of 1.5 mg/kg beta-carotene. A literature review study showed that carotenoids played a role in the mechanism of ROS, inflammation, neovascularization, and neurodegeneration in both diabetes mellitus patients and diabetic test animals. Furthermore, a dose of carotenoids in the form of astaxanthin of 3 mg/kgBW in rats significantly protects the retina in inhibiting neurodegenerative diseases [29,30]. The effect of alpha tocopherol in the diabetic rat model by Ichsan et al. was reported to function as a retinoprotective agent when given a single dose of 15 mg for 14 days (pre- and post-alloxan induction), obtaining an average photoreceptor cell density of 752 ± 190 cells and a retinal ganglion cell density of 27 ± 2 cells [22].

The result of this study showed a retinoprotective effect on photoreceptor cells and ganglion cells in treatment group 3. This group was given 1 mL of *buah merah* extract with a combination of active compounds of 12 mg total carotenoids, 10 mg tocopherol, 3.581 mg beta-carotene, 1.460 mg B-cryptoxanthin, and 1.368 mg alpha tocopherol. A previous study on diabetic rats given a combination of antioxidants also showed an antioxidant effect that protected the retina of diabetic rats [25]. In various studies that assessed retinal layer using OCT, the inner retinal layer, including nerve fibers, ganglion cells, and the plexiform experienced a decrease along with the duration of RD incident. Caspase-3 is an executor that has been widely examined in relation to the apoptosis process in the neural retina.

Previous studies have reported that red fruit oil supplementation may not always provide protective effects, as seen in experimental models where it failed to prevent oxidative stress, raising concerns about its potential limitations and safety profile; however, in our study, no overt adverse effects were observed during the treatment period, with animals showing no behavioral changes, weight loss, or gastrointestinal symptoms, suggesting that the supplementation was well tolerated under our experimental conditions [19,31,32].

The unique bioactive components of this extract—particularly β-cryptoxanthin, tocopherol, and polyunsaturated fatty acids—are known to modulate oxidative stress and apoptotic signaling in retinal tissue. Specifically, these compounds may inhibit the caspase-3 activation pathway, reducing DNA fragmentation and apoptotic cell death among photoreceptor and ganglion cells. In addition, the antioxidant properties of β-cryptoxanthin and tocopherol contribute to the stabilization of mitochondrial membranes and preservation of retinal neuron integrity under hyperglycemic conditions. This mechanistic explanation aligns with recent studies demonstrating that carotenoid-rich and antioxidant extracts can suppress apoptosis through regulation of Bcl-2/Bax expression and attenuation of ROS-mediated damage in diabetic retina models (see updated references. These findings suggest that Pandanus conoideus extract exerts a dual protective role—both anti-oxidative and anti-apoptotic—which may contribute to the preservation of retinal structure and function in diabetic conditions [18].

In this study, Alloxan-induced diabetic rats showed that apoptosis had occurred, evidenced by the expression of caspase-3 in photoreceptor cells and ganglion cells in the diabetic rat model for approximately 14 days of observation. This was also found in a study conducted on an STZ-induced diabetic rat model to assess apoptosis that occurred starting from 1 week of observation. The result showed that the comparison of caspase-3 expression in photoreceptor cells in treatment groups 3, 4, and 5 was not significantly different between the normal and diabetic groups. Groups 3 and 4 affected the photoreceptor cells of experimental rats, with caspase-3 expression values in photoreceptor cells similar to normal controls, while group 5 had no effect. Caspase-3 expression in ganglion cells showed that all treatment groups had different values from the normal control group. Therefore, at the retinal ganglion cell level, the effect of this *buah merah* extract did not inhibit retinal ganglion cell apoptosis. The administration of *buah merah* extract containing alpha tocopherol and carotenoids has the potential to protect photoreceptor cells but not ganglion cells. Another study explained the effect of alpha tocopherol on a group of rats given a dose of 15 mg alpha, approaching the normal group’s caspase-3 expression value [22]. A study conducted for 21 days showed that the dose range of *buah merah* extract should represent the minimum and maximum with the same interval. The minimum dose in experimental rats was 4 g/kgBW, and the maximum was 68 g/kgBW. Another study showed the hypoglycemic effect of *buah merah* extract in Alloxan-induced diabetic rats (14 mg, IV). The result showed that doses ranging from 0.12 mL to 0.48 mL were safe in experimental animals, with 0.12 mL exhibiting a significant hypoglycemic effect [28].

Kaur N. et al. (2017) reported that doses of 1.35 mL/kg BW and 5.4 mL/kg BW did not impair kidney function in STZ-induced rats [33]. Meanwhile, reports on diabetic rats given 15 mg/kgBW of alpha tocopherol compounds have shown a retinoprotective effect [22]. In this study, the dose range given was 1–2 mL. The results showed that a 1 mL dose of *buah merah* extract, containing 12 mg of total carotenoids, 10 mg of total tocopherol, 1.348 mg of alpha-tocopherol, and 3.4 mg of beta-carotene, protected photoreceptor and retinal ganglion cells from apoptosis in diabetic rats. An active compound in natural plants can act as an antioxidant when it fights the effects of free radicals on a disease [30]. However, there is still controversy regarding the existence of a pro-oxidant effect. In this case, when an active compound is given in excessive amounts, the free radical process increases. According to Homer et al. (2020), the pro-oxidant effects of alpha tocopherol and beta carotene lead to increased ROS in vitro studies. Another report showed that the administration of *buah merah* oil doses could cause increased ROS in experimental animals due to the combined work of several antioxidants as pro-oxidants by inducing the Fenton reaction and the Haber-Weiss reaction. This increases the production of hydroxyl radicals and reduces antioxidant levels in the blood [34]. A study by de Oliveira, B.F. (2013) found that a combination of antioxidants, consisting of 1.6 μM ascorbic acid, 0.82 μM alpha-tocopherol, and 0.016 μM beta-carotene, induced free radical production in vitro in type 1 diabetes mellitus patients aged 20–39 years [35]. This may be due to the doses exceeding the optimal levels of 12 mg total carotenoids, 10 mg total tocopherol, 1.348 mg alpha-tocopherol, and 3.4 mg beta-carotene, rendering the 1.5 mL and 2 mL doses ineffective in protecting retinal cells in diabetic rats [36].

Safety factors will remain a major concern in the use of natural plants as supplements for DR. Therefore, the advantages of this study include relevance to clinical situations related to the prevention and treatment of DR. The results are expected to have good potential clinical impacts. This study used a detailed and methodological experimental design to evaluate the effects of *buah merah* extract on the retina of the diabetic rat model, including clear control and treatment groups. A histological analysis was used to evaluate cellular changes in the retina, and important qualitative and quantitative data were provided. The number of experimental animal samples was sufficient to provide results that could be analyzed statistically. However, there are limitations, including the use of experimental rats as a diabetic model cannot fully represent the pathomechanism of DR in humans. Although animal models are useful, the results of this study should be applied with caution to human clinical conditions. The 14-day treatment duration may also be a limitation because the observation of long-term therapeutic effects of *buah merah* extract is not yet fully understood. The assessment of density and apoptosis depends significantly on histopathological methods that may still override molecular or biochemical data.

There has been increasing interest in the field of study related to natural plants that are eventually formulated as supplements. Regarding side effects and drug interactions, Zhao et al. reported that some plants did not show toxic effects in vivo or in vitro. However, cellular and animal model studies may not fully predict toxicological outcomes in humans [37]. In our study, the application of *buah merah* extract represents a groundbreaking step in expanding the scientific repertoire of ocular therapeutics. By focusing on its protective effects against retinal cell apoptosis, particularly in photoreceptors and retinal ganglion cells, this research provides novel insights into its role as a potential neuroprotective and cytoprotective agent in the eye. Such findings not only validate the empirical use of *buah merah* in traditional medicine but also open a new frontier in ophthalmic research, underscoring its promise as a natural intervention for vision preservation and retinal health. These findings highlight the importance of dose optimization and mechanistic studies to clarify the balance between neuroprotection and oxidative signaling in diabetic retinal models.

## 5. Conclusions

In conclusion, this study provides an overview of the effects of administering *buah merah* extract on the diabetic rat model. The results of this study show that natural plant ingredients, such as *buah merah*, have the potential to be retinoprotective in the diabetic rat model, even though hyperglycemia has not been resolved. There is a possibility that the antioxidant effect of *buah merah* extract may include additional pathways beyond inhibiting oxidative stress in DR. Future research also should include longer observation periods and molecular assessments of oxidative stress and apoptosis-related markers to confirm the neuroprotective mechanisms of *buah merah* extract. Since *buah merah* has been traditionally used by Indigenous people, supported by experiential knowledge, translation into clinical pilot trials in humans is also recommended to validate its therapeutic potential.

## Figures and Tables

**Table 1:** Blood glucose level of the experimental animal.

Blood Glucose Level (mg/dL)						
Group	Pre-Alloxan (Mean ± SD)	Post-Alloxan (Mean ± SD)	*p*-Value	Post-Alloxan (Mean ± SD)	Pre-Termination (Mean ± SD)	*p*-Value
1	103.50 ± 10.03	N/A	**-**	N/A	107.00 ± 3.90	**-**
2	109.50 ± 16.87	220.17 ± 68.64	**0.038**	220.17 ± 68.64	418.83 ± 144.51	**0.012**
3	106.83 ± 15.05	307.33 ± 97.66	**<0.001**	307.33 ± 97.66	371.83 ± 61.40	**0.031**
4	113.50 ± 15.28	341.50 ± 128.10	**0.010**	341.50 ± 128.10	375.83 ± 66.79	**0.182**
5	106.40 ± 12.59	453.80 ± 65.40	**<0.001**	453.80 ± 65.40	374.00 ± 51.16	**<0.001**

Groups = 1: normal control; 2: diabetic control; 3: diabetic + 1 mL extract; 4: diabetic + 1.5 mL extract; 5: diabetic + 2 mL extract.

**Table 2:** Density of photoreceptor cells and ganglion cells in experimental animals.

Group	Photoreceptor Cell Density (Mean ± SD)	*p*-Value	Retinal Ganglion Cell Density (Mean ± SD)	*p*-Value
1	787.97 ± 18.77	* 0.001	22.77 ± 2.63	* 0.001
2	572.93 ± 45.40		13.20 ± 2.31	
3	722.52 ± 147.56		18.73 ± 5.61	
4	605.03 ± 77.46		14.86 ± 3.28	
5	604.28 ± 75.23		13.84 ± 3.13	

* One-Way ANOVA test is significant with a *p*-value <0.05. Groups = 1: normal control; 2: diabetic control; 3: diabetic + 1 mL extract; 4: diabetic + 1.5 mL extract; 5: diabetic + 2 mL extract.

**Table 3:** Caspase-3 expression in photoreceptor cells of experimental animals.

Group	Caspase-3 Expression in Photoreceptor Cells (n)	*p*-Value
1	*Negative*: 5	**0.020**
	*Low*: 1	
	*High*: 0	
2	*Negative*: 0	
	*Low*: 4	
	*High*: 2	
3	*Negative*: 3	
	*Low*: 3	
	*High*: 0	
4	*Negative*: 2	
	*Low*: 4	
	*High*: 0	
5	*Negative*: 1	
	*Low*: 4	
	*High*: 0	

The Kruskal–Wallis test is significant with a *p*-value <0.05. n = subject (rats) (groups = 1: normal control; 2: diabetic control; 3: diabetic + 1 mL extract; 4: diabetic + 1.5 mL extract; 5: diabetic + 2 mL extract).

**Table 4:** Expression of caspase-3 in ganglion cells in experimental animals.

Group	Caspase-3 Expression in Retinal Ganglion Cells (n)	*p*-Value
1	23.50 ± 3.51	0.001 *
2	46.33 ± 6.05	
3	37.50 ± 5.01	
4	38.33 ± 9.67	
5	41.60 ± 4.67	

* The One-Way ANOVA test is significant with a *p*-value <0.05 (groups = 1: normal control; 2: diabetic control; 3: diabetic + 1 mL extract; 4: diabetic + 1.5 mL extract; 5: diabetic + 2 mL extract).

## Data Availability

The original contributions presented in this study are included in the article. Further inquiries can be directed to the corresponding author.
